# Case Report: Ulcerative breast cancer occurring 20 years after breast augmentation with Amazingel injection

**DOI:** 10.3389/fonc.2026.1952422

**Published:** 2026-09-03

**Authors:** Tian Li, Yun Tong, Mengyao Luo

**Affiliations:** 1 Department of Plastic Surgery, Affiliated Jinhua Hospital, Zhejiang University School of Medicine, Jinhua Municipal Central Hospital., Jinhua, Zhejiang, China; 2 Department of Obstetrics, Hangzhou Women’s Hospital, Hangzhou, Zhejiang, China

**Keywords:** Amazingel, breast cancer, case report, injectable breast augmentation, invasive ductal carcinoma, ulcerative type

## Abstract

**Background:**

Polyacrylamide hydrogels (PAAG), commercially known as Amazingel, have been widely used for injectable breast augmentation in China since the early 2000s. Because PAAG is non-degradable, long-term retention in the body may lead to complications, including infection, granuloma formation, and induration. Concerns regarding its potential long-term carcinogenicity have also emerged. However, breast cancer developing after PAAG injection has rarely been reported, and cases occurring more than 20 years after injection are exceptionally rare. Previous reports have not described ulcerative presentations or detailed molecular subtypes.

**Case presentation:**

We report a case of unilateral ulcerative breast cancer that developed 20 years after PAAG injection for breast augmentation. Following removal of the injected PAAG material and surgical debridement, the infection was controlled, and histopathological examination confirmed invasive ductal carcinoma. Immunohistochemistry demonstrated ER (3+), PR (3+), HER2 (2+), whereas Fluorescence *in situ* hybridization (FISH) testing suggested a HER2-negative status. Endocrine therapy was subsequently initiated.

**Conclusions:**

To our knowledge, this is the first reported case of ulcerative breast cancer occurring 20 years after PAAG injection for breast augmentation. The tumor exhibited a molecular profile of particular interest, characterized by robust hormone-receptor positivity and low HER2 expression. Ultra-long-term retention of PAAG may show a temporal association with breast cancer development, highlighting the need for long-term clinical surveillance decades after injection.

## 1 Introduction

Amazingel, also known as polyacrylamide hydrogel (PAAG), is a hydrophilic gel composed of 5% polyacrylamide and 95% water (1). Owing to its rapid onset of action and favorable cosmetic outcomes, PAAG injection for breast augmentation became widely used among Chinese women in the early 2000s. It has been estimated that more than 300,000 women underwent PAAG injection for breast augmentation.

However, as its clinical use became more widespread, concerns regarding the long-term safety of PAAG gradually emerged. As a non-degradable filler, PAAG is not absorbed after implantation and can remain in breast tissue indefinitely. Clinical studies have reported a range of complications, including infection, gel migration, breast deformity, induration, granuloma formation, and fistula development (2). More severe complications include persistent infection, skin ulceration, delayed wound healing at the injection site, and, in rare cases, breast cancer (3). *In vitro* studies have suggested that the acrylamide monomers released during PAAG degradation can bind to guanine residues in DNA to form adducts, resulting in DNA damage and gene mutations. These findings provide mechanistic evidence supporting the potential carcinogenicity of PAAG (4, 5).

On April 30, 2006, the State Food and Drug Administration of China officially revoked the medical device registration certificate for PAAG, thereby prohibiting its production, sale, and clinical use. Nevertheless, the material remained in the bodies of patients who had previously undergone PAAG injection, and long-term complications have continued to emerge many years later. Although isolated cases of breast cancer following PAAG injection for breast augmentation have been reported, advanced breast cancer presenting initially with skin ulceration more than 20 years after injection is exceptionally rare. Here, we report a case of ulcerative breast cancer that developed 20 years after PAAG injection for breast augmentation. This case highlights the need for continued long-term surveillance of patients with retained PAAG and adds to the limited body of evidence concerning long-term clinical outcomes and clinical management in this patient population.

## 2 Case presentation

**Figure 1:**
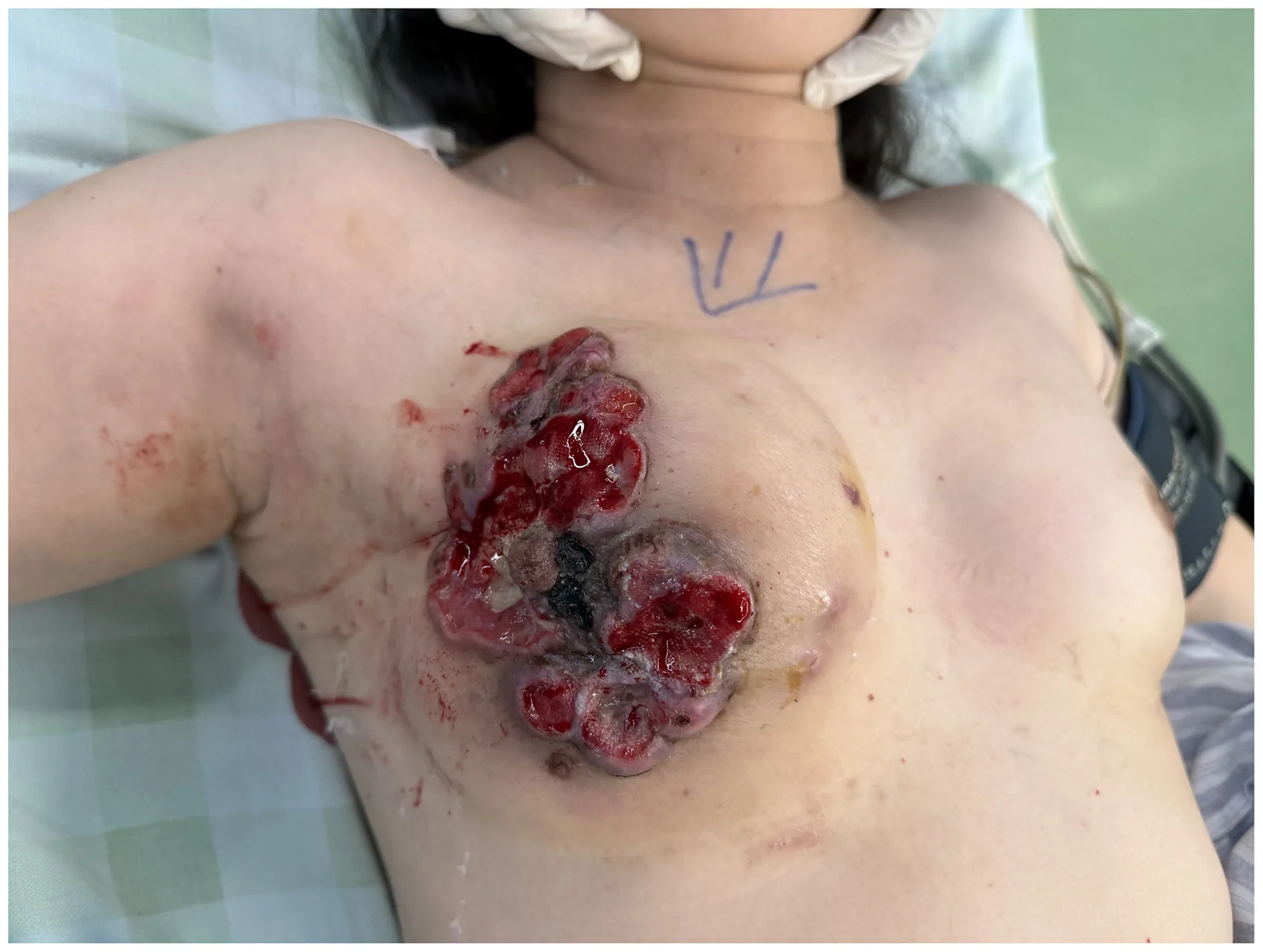
Comparison of the patient’s left and right breasts before surgery. The right breast showed ulcerative skin changes, while the left breast demonstrated intact skin and no pathological skin changes.

A 53-year-old woman underwent PAAG injection for breast augmentation 20 years previously. Fifteen years earlier, she underwent removal of the left-sided PAAG filler because of acute mastitis. Two years before presentation, she developed a sinus tract in the right breast with persistent yellow fluid discharge. Three months before admission, she presented to our plastic surgery department with skin ulceration and necrosis of the right breast accompanied by erythema, swelling, warmth, and pain. A comparison of the left and right breasts is shown in **Figure 1**.

**Figure 2:**
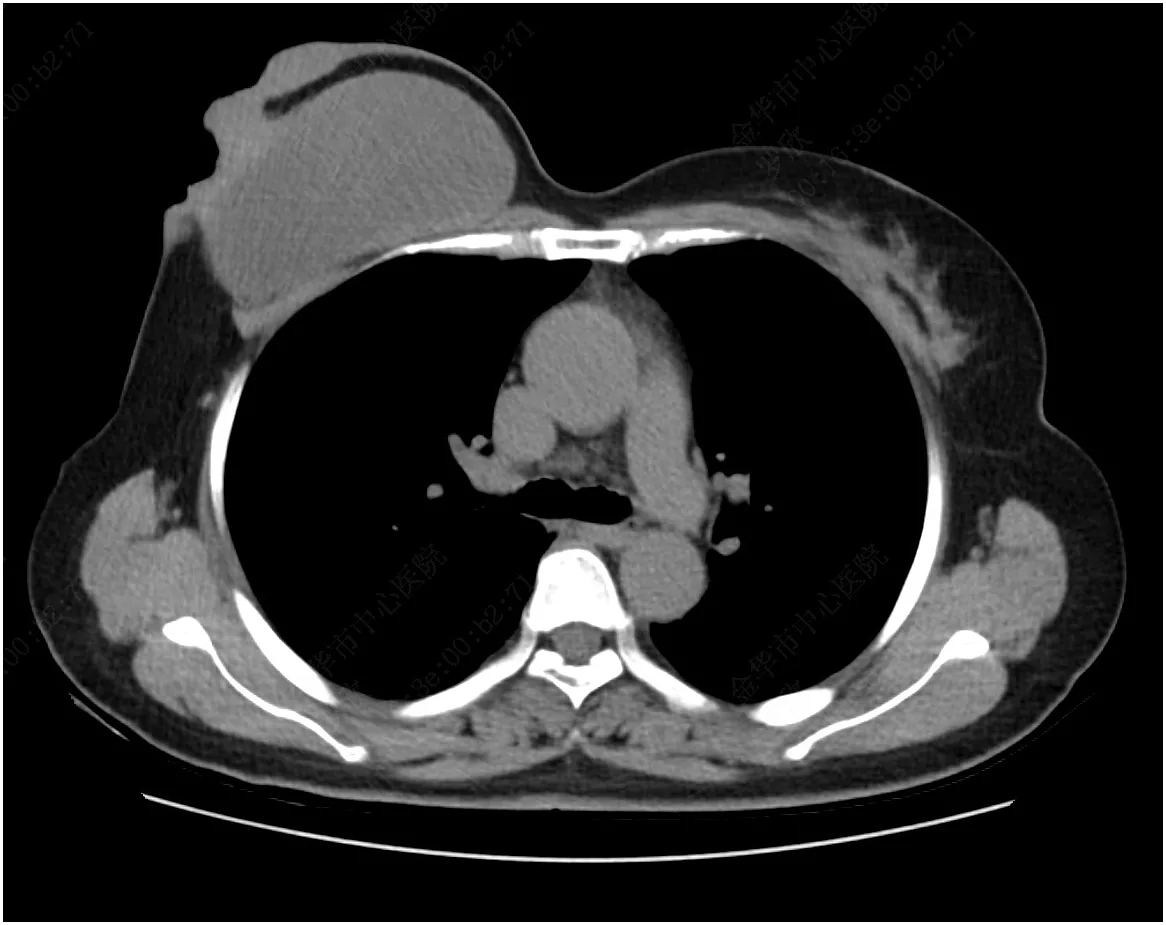
Computed tomography scan showing injected PAAG material within the right breast after augmentation, with irregular thickening of the surrounding soft tissues, multiple enlarged bilateral axillary lymph nodes, and bony destruction of the L1 vertebra.

The patient’s body mass index was 22.3 kg/m^2^. Laboratory investigations revealed a white blood cell count of 8.39 × 10^9^/L, hemoglobin level of 125 g/L, platelet count of 368 × 10^9^/L, high-sensitivity C-reactive protein level of 20.15 mg/L, procalcitonin level of 0.08 ng/mL, carcinoembryonic antigen level of 8.31 ng/mL, and CA15–3 level of 74.31 U/mL. These findings indicated elevated inflammatory markers and abnormal tumor marker levels. Preoperative chest computed tomography (**Figure 2**) demonstrated post-augmentation changes in the right breast, with irregular thickening of the surrounding soft tissue, multiple enlarged bilateral axillary lymph nodes, and destruction of the L1 vertebral body.

Following multidisciplinary discussion, a biopsy was recommended to establish the diagnosis. A 14-gauge core-needle biopsy was performed in our outpatient department, with sampling taken from the ulcer margin for pathological examination. Histopathological examination revealed scattered histiocytes, clusters of ductal epithelial-like cells, and sheets of basophilic amorphous material consistent with filler material. After discussion with the patient and her family, removal of the PAAG filler with surgical debridement was performed to control the severe local infection while obtaining definitive pathological specimens.

**Figure 3:**
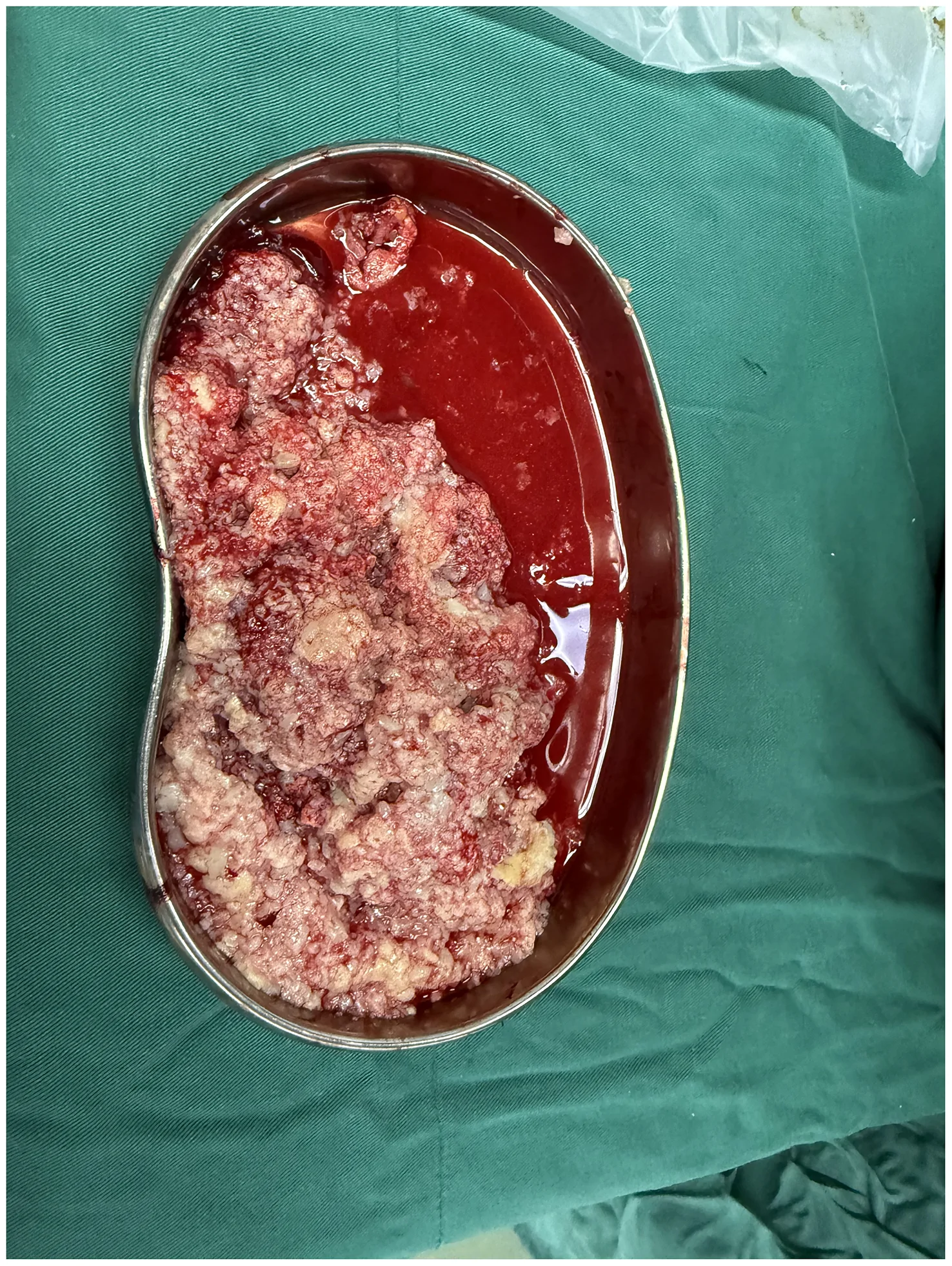
Approximately 300 mL of jelly-like filler was removed during the operation.

**Figure 4:**
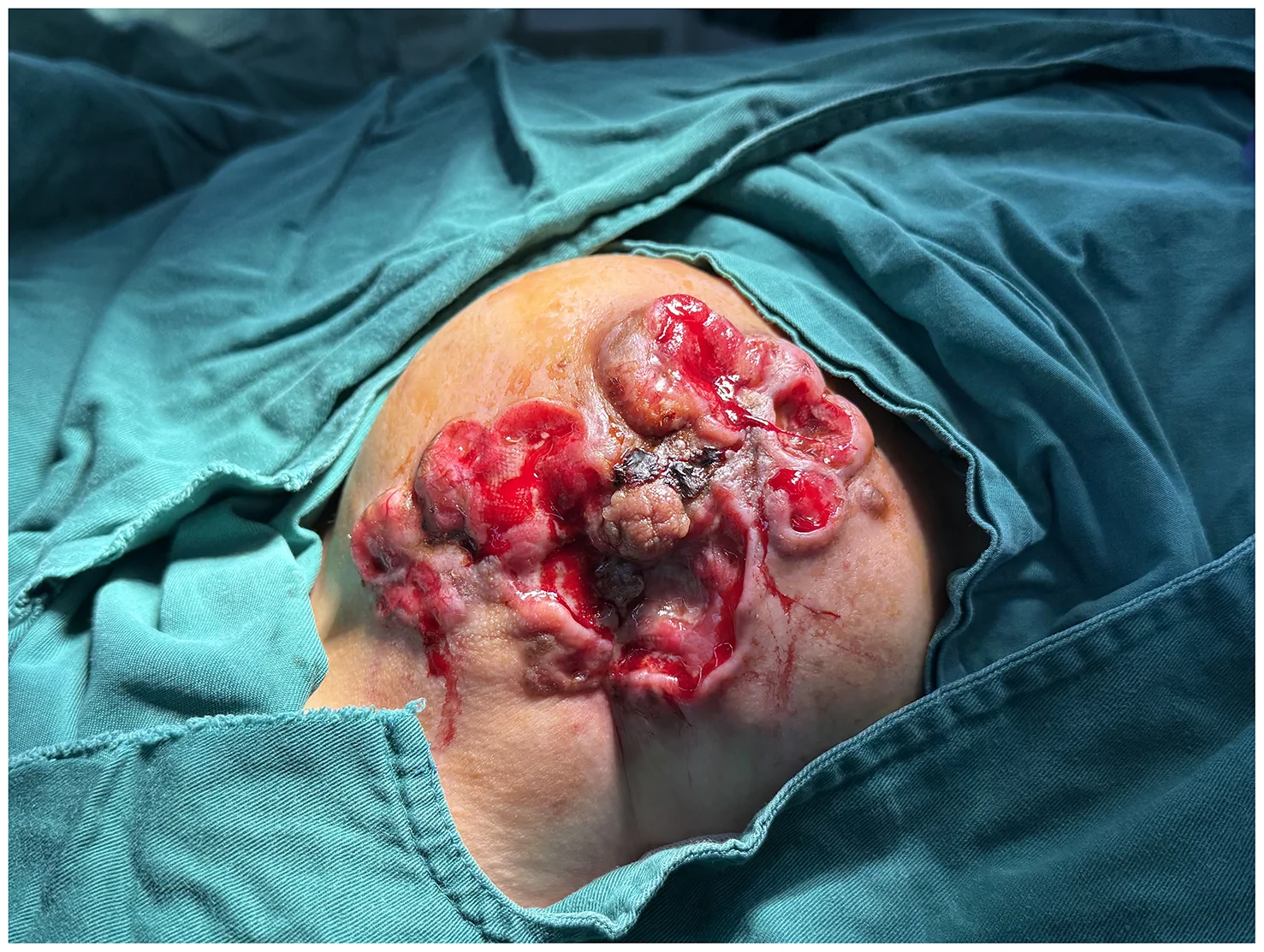
Surgical wound during the operation.

Under local infiltration anesthesia, the skin was incised to the subcutaneous layer, and electrocautery was used for hemostasis and tissue dissection. Yellow, viscous, granular material was observed beneath the breast. A large amount of jelly-like filler adherent to the surrounding tissues was removed, relieving compression of the right breast. Approximately 300 mL of filler was evacuated (**Figure 3**). Necrotic tissue from the resection site was submitted for histopathological examination. The surgical wound is shown in **Figure 4**. The wound was thoroughly irrigated with copious saline and diluted iodophor solution, packed with Vaseline gauze, and dressed, with delayed wound closure planned. The procedure lasted approximately 75 minutes, with an estimated blood loss of 50 mL. No blood transfusion was required.

**Figure 5:**
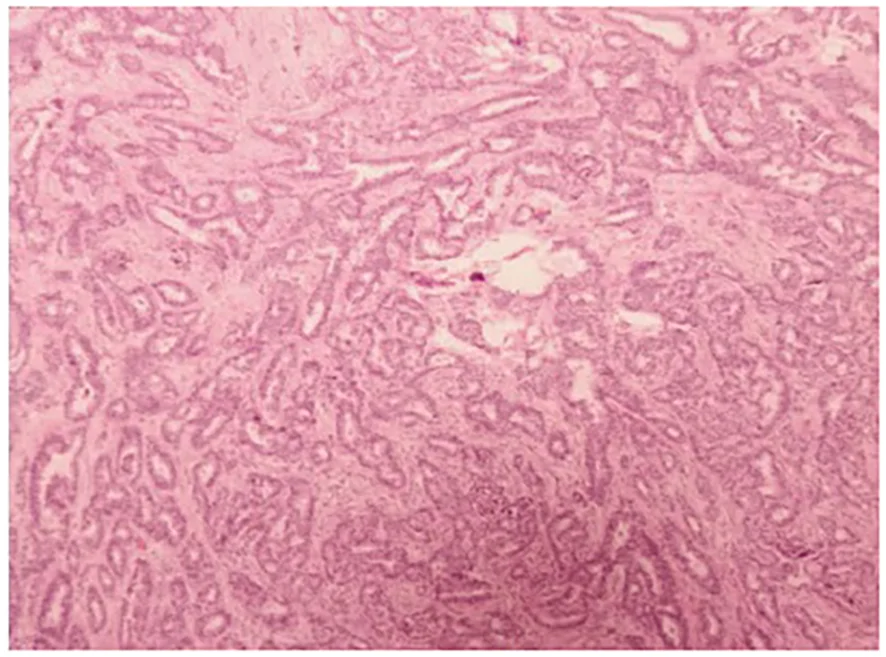
Postoperative pathological result showing invasive ductal carcinoma involving the skin.

**Figure 6:**
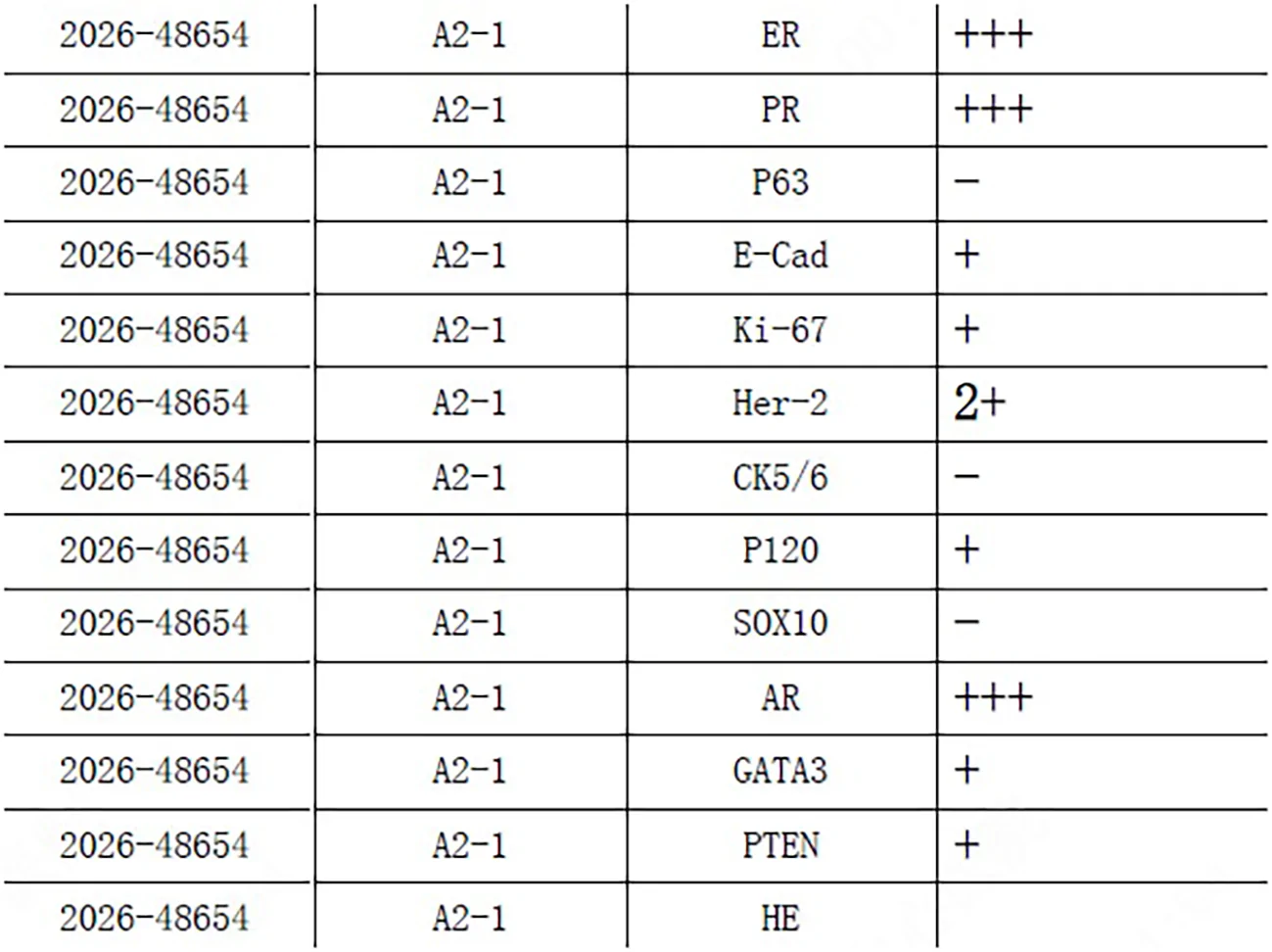
Immunohistochemistry showing ER (3+), PR (3+), and HER2 (2+) statuses. The FISH test indicated HER2 negative status.

Postoperative histopathological examination confirmed moderately differentiated invasive ductal carcinoma with direct dermal invasion (**Figure 5**). Multinucleated giant cells and foreign-body granulomatous inflammation were observed surrounding PAAG lacunar spaces within the breast parenchyma. These PAAG-associated inflammatory changes were spatially adjacent to but not directly intermixed with tumor nests, indicating co-existing foreign-body reaction in the setting of long-term PAAG retention rather than direct inflammatory-tumor interpenetration. Immunohistochemistry demonstrated ER (3+), PR (3+), and HER2 (2+) expression, whereas Fluorescence *in situ* hybridization (FISH) confirmed HER2-negative status (**Figure 6**).

**Figure 7:**
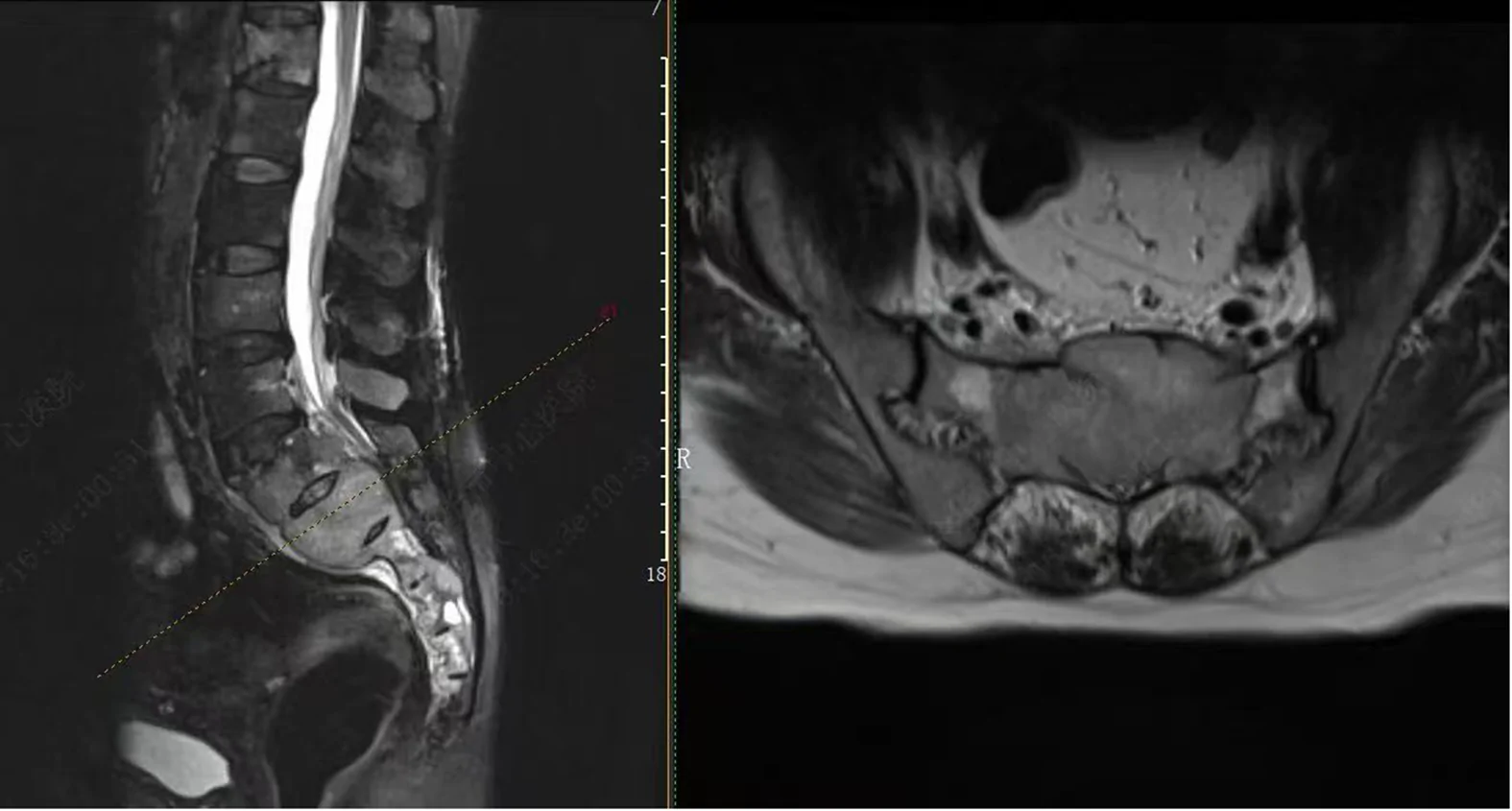
Spinal MRI of the patient demonstrated multiple patchy ill-defined abnormal signals in the thoracic, lumbar and sacrococcygeal vertebrae, consistent with bone metastasis.

Pre-operative chest CT revealed lumbar vertebral bone destruction. Spinal MRI was subsequently performed for definitive diagnosis, which demonstrated multiple patchy, ill-defined abnormal signals within the thoracic, lumbar, and sacrococcygeal vertebrae, consistent with bone metastasis (**Figure 7**). Ultrasound demonstrated bilateral axillary lymphadenopathy; the right axillary lymph nodes exhibited morphological features suspicious for malignancy, including obliterated fatty hilum, cortical thickening >3 mm, and rounded morphology. Ultrasound-guided fine-needle aspiration biopsy of the right axillary lymph node confirmed metastatic carcinoma. The contralateral axillary lymph nodes were interpreted as reactive changes and were not biopsied. Integrating imaging findings, biopsy results, and physical-examination evidence of chest-wall skin ulceration, the patient was assigned a clinical stage of cT4N1M1.

After transfer to the breast surgery department, surgical resection was considered unlikely to provide substantial benefit because of the extensive tumor burden and concurrent infection. Given the strong hormone receptor positivity, the multidisciplinary team recommended endocrine therapy, together with regular wound care and anti-infective treatment. Intra-operative cultures isolated *Acinetobacter baumannii* and *Staphylococcus aureus*, and no fungi were detected. After debridement, the patient received 10-day therapy with intravenous ceftriaxone 2 g once daily combined with topical silver sulfadiazine. Decreased wound exudate, resolution of peripheral erythema, negative repeat wound culture, and normalized C-reactive protein confirmed that the infection was controlled.

The patient subsequently initiated endocrine therapy combined with a Cyclin-Dependent Kinase 4 and 6 (CDK4/6) inhibitor. The treatment regimen consisted of letrozole 2.5 mg orally once daily and ribociclib 600 mg orally once daily for 21 days followed by a 7-day treatment-free interval in each 28-day cycle. To reduce skeletal-related events, denosumab 120 mg was administered subcutaneously every 4 weeks. At the time of writing, the patient remains on this treatment regimen, is in generally good condition, and continues regular follow-up.

After three months of letrozole plus ribociclib treatment, the patient showed marked improvement in the chest-wall wound with reduced exudate. C-reactive protein returned to the normal range, indicating adequate infection control. Apart from occasional nausea, no other notable treatment-related adverse events were observed.

The study was approved by the Ethics Committee of Jinhua Central Hospital, and the patient provided written informed consent. The study was conducted in accordance with the Declaration of Helsinki (revised in 2013).

## 3 Discussion

We report a rare case with important clinical implications in which ulcerative invasive ductal carcinoma developed 20 years after PAAG injection for breast augmentation. The noteworthy features of this case lie not only in its exceptionally long latency period and aggressive clinical presentation characterized by skin ulceration but also in its molecular profile of particular interest, with robust hormone-receptor positivity (ER 3+, PR 3+) and HR-positive/HER2-low (IHC 2+, FISH-negative). To our knowledge, this is the first reported case of ulcerative breast cancer occurring 20 years after PAAG injection with this molecular profile.

Previous reports have described breast cancer occurring 5–15 years after PAAG injection. For example, a study summarizing 135 cases of PAAG-related complications identified only four cases of breast cancer (6), whereas another clinicopathological analysis of 90 patients reported only two subsequent malignancies (4). The longest latency period previously reported in the English-language literature was 20 years; however, that case primarily presented with imaging findings mimicking malignancy secondary to PAAG-related complications and was not histopathologically confirmed as invasive breast cancer (7). In contrast, our patient presented with skin ulceration and histopathologically confirmed invasive ductal carcinoma 20 years after PAAG injection, highlighting a temporal association between ultra-long-term PAAG retention and breast cancer development. An additional distinguishing feature of this case is its molecular profile. Most previously reported cases of breast cancer following PAAG injection have involved biologically aggressive subtypes, including HER2-positive or triple-negative disease, whereas our patient demonstrated strong hormone receptor positivity with low HER2 expression. This case expands the limited spectrum of reported molecular profiles among breast cancers occurring after PAAG injection and suggests that any potential carcinogenic effects of PAAG may not be restricted to a single molecular subtype. Instead, long-term chronic stimulation by PAAG may contribute to malignant transformation through different molecular pathways.

The mechanisms hypothesized to associate PAAG with malignant transformation remain incompletely understood, with prevailing hypotheses focusing on chemical toxicity and chronic foreign-body-mediated physical stimulation. From a chemical standpoint, long-term degradation of PAAG may release genotoxic acrylamide monomers. Acrylamide is categorized by the International Agency for Research on Cancer as a Group 2A (“probably carcinogenic to humans”) agent. Upon metabolic conversion to propylene oxide by cytochrome P450 2E1, acrylamide forms DNA adducts that drive point mutations and genomic instability (5). From a physical perspective, PAAG functions as a non-degradable foreign body that triggers chronic granulomatous inflammation, fibrosis, and tissue necrosis. This inflammatory microenvironment accumulates reactive oxygen species, inflammatory cytokines including IL-6 and TNF-α, and growth factors capable of promoting aberrant cellular proliferation and survival via activation of signaling cascades such as NF-κB and STAT3 (8, 9). We acknowledge that these mechanistic hypotheses, although derived from prior literature, remain speculative when interpreted in the setting of a single case and cannot be invoked to explain the distinct molecular features of the tumor described herein. Potential connections between chronic inflammatory mediators, estrogen-related signaling, epigenetic alterations, and the outgrowth of hormone-receptor-positive malignant clones have been postulated in pre-clinical investigations; nevertheless, such associations are unproven in human clinical cases of PAAG exposure. While HER2 amplification was ruled out by FISH, the low-HER2 phenotype suggests that tumor progression may rely on alternative signaling pathways, which may represent actionable therapeutic targets.

This case provides several important clinical insights. First, the exceptionally long latency period suggests that the potential adverse effects of retained PAAG may extend well beyond the first decade after injection. Although recommendations for long-term surveillance of patients with retained PAAG vary, this case supports consideration of prolonged breast surveillance, including breast ultrasonography, mammography, and magnetic resonance imaging when clinically indicated (10). Second, ulcerative breast lesions developing after PAAG injection may be misinterpreted as chronic infection, foreign body reaction, or skin necrosis, potentially delaying the diagnosis of an underlying malignancy (11). Therefore, breast cancer should remain in the differential diagnosis in patients with previous PAAG injection who develop persistent ulceration or nonhealing wounds, and prompt tissue biopsy should be considered when clinically appropriate. Third, the molecular profile observed in this case supported treatment with endocrine therapy. In addition, the HER2-low phenotype may have therapeutic implications should disease progression occur, as HER2-directed Antibody-Drug Conjugate (ADC) drugs, such as trastuzumab deruxtecan, have demonstrated clinical benefit in selected patients with HER2-low breast cancer (12, 13). This case further highlights the importance of individualized treatment based on molecular typing rather than assumptions regarding the biological behavior of malignancies occurring after PAAG injection.

Several limitations should be acknowledged. As a single case report, this study cannot establish a causal relationship between PAAG injection and breast cancer development, and the temporal association observed may be coincidental. Furthermore, quantitative analysis of PAAG degradation products within the tumor tissue was not performed, and molecular characterization of the inflammatory microenvironment, including cytokine profiling and genomic analyses, was unavailable. Nevertheless, this case represents one of the longest documented intervals between PAAG injection and histopathologically confirmed ulcerative invasive breast cancer. Future multicenter registries and prospective studies are warranted to clarify the true incidence, latency, clinicopathological characteristics, and potential mechanisms underlying breast cancer occurring after PAAG injection.

## 4 Patient perspective

The patient is a 53-year-old woman who experienced a journey from shock and self-blame to active coping after being diagnosed with breast cancer. She recalled that when she underwent PAAG injection augmentation mammoplasty 20 years ago, she was completely unaware of any long-term risks, choosing the procedure simply because it was “simple and required a short recovery.” Over the following two decades, she never underwent any dedicated breast screening, attributing occasional breast discomfort to “normal changes.” It was not until skin ulceration appeared and persisted that she sought medical attention, still believing it to be an infection or foreign-body reaction, never suspecting malignancy.

Upon diagnosis of locally advanced breast cancer with subsequent bone metastasis, she experienced a period of depression. However, after her physicians thoroughly explained her molecular subtype (hormone receptor-positive, HR-positive/HER2-low) and the availability of targeted endocrine combination therapy, she regained confidence.

The patient hopes that by sharing her story, she can alert all women who have received PAAG injections: even if many years have passed and no symptoms are present, annual breast imaging surveillance is essential; any skin ulceration or palpable mass should prompt immediate medical evaluation without delay. She expresses gratitude to her medical team and looks forward to more novel agents for HER2-low breast cancer, which may offer prolonged survival for patients like her.

## Data Availability

The raw data supporting the conclusions of this article will be made available by the authors, without undue reservation.
